# 

**Published:** 1875-11

**Authors:** 


### TABLE OF CONTENTS.

ORIGINAL COMMUNICATIONS.

Duodenitis. By A. L. Knight, M. D., West Columbia, West Virginia........ 641
Ergot. By T. Curtis Smith, Middleport, Ohio................................ 646
Is Chorea a Sequence of Rheumatism? By J. C. Bishop, M. D., Middleport, Ohio.............................................. 653
Musca Volitentes. By John Coston, M. D., Bowdon, Georgia.............. 656

SELECTIONS.

The Treatment of True Membranous Croup. By W. H. Vail, M. D., Cornwall-on-Hudson, N. Y.............................................. 659
Case of Double Vagina and Uterus. By A. E. Hoadley, M. D., Chicago..... 661
Generators of Uric Acid in the Organism................................ 663
Hepatic Colic Cured by Chloroform. By Q. C. Smith, M. D.............. 665
Solubility of Biliary Calculi Within the Gall-Bladder. By Ralph S. Goodwin, M. D., of Thomaston, Conn.............................................. 667
On Hypodermic Injection................................................ 671
Ergot as an Anti-Galactagogue.......................................... 672

EXTRACTS AND GLEANINGS.

Menstruation Not a Physiological Process—673. Respiratory Percussion—675. Scarless Eradication of Nævi—677. On Croton-Chloral Hydrate—679. Bromide of Ammonium in Excessive Menses—680. Luxation of the Penis—682. Influence of Anæsthetics upon the Sexual Impressions of Females—683. Common Colds—684. Mercurial Baths in Syphilis—685. Diminution of the Uterus after Delivery—687. Cause and Prevention of Typhoid Fever in Schools—687. Successful Ovariotomy Performed Under Numerous Difficulties; Hot Baths—688. Ergot—689. Secondary Syphilis—689. Local Treatment of Cancer—690. New M[illegible]rial for Fixed Dressings—691. Subcutaneous Injections of Strychnia in [illegible]continence of Urine—692. Acid Tannate of Iron in Diphtheria—693. Lingering Labor from Rigidity of the Soft Structures—694. Ether or Chloroform?—695. Gargle for Sore Throat—695. Ointment fer Sycosis—695. Gelseminum in Odontalgia and Facial Neuralgia—695. The Danger of Cantharides—696. Medicated Milk—696. Catarrhal Affections—696. Ergot in the Treatment of Insanity—696. Solubility of Salicylic Acid—697. Treatment of Ozœma—687. Solution of Morphia for Hypodermic Injections—697. Syrup of Acacia—697. Diagnosis of Ovarian Diseases—698. Ascarides Treated by Enemata of Cod-Liver Oil—698. Remedy for Chronic Hoarseness—698.

EDITORIAL AND MISCELLANEOUS.

Delinquent Subscribers................................................. 699
The Warren Prize....................................................... 699
Women in Medicine, Law and Theology.................................... 700

